# Toll-Like Receptors, Their Ligands, and Atherosclerosis

**DOI:** 10.1100/tsw.2011.36

**Published:** 2011-02-14

**Authors:** Conrad P. Hodgkinson, Shu Ye

**Affiliations:** ^1^ Clinical Pharmacology, William Harvey Research Institute, Charterhouse Square, London, UK; ^2^ Department of Medicine, Duke University Medical Center and Mandel Center for Hypertension and Atherosclerosis Research, Durham, NC, USA

**Keywords:** Toll-like receptors, innate immunity, atherosclerosis, heart disease

## Abstract

Atherosclerosis is a disease characterized by inflammation in the arterial wall. Atherogenesis is dependent on the innate immune response involving activation of Toll-like receptors (TLRs) and the expression of inflammatory proteins. TLRs, which recognize various pathogen-associated molecular patterns, are expressed in various cell types within the atherosclerotic plaque. Microbial agents are associated with an increased risk of atherosclerosis and this is, in part, due to activation of TLRs. Recently considerable evidence has been provided suggesting that endogenous proteins promote atherosclerosis by binding to TLRs. In this review, we describe the role of TLRs in atherosclerosis with particular emphasis on those atherogenic endogenous proteins that have been implicated as TLR ligands.

## INTRODUCTION

Coronary artery disease predominantly results from atherosclerosis, a process involving the buildup of modified lipids, cholesterol, and cellular debris within the inner lining of the arterial wall.

Atherosclerosis begins with damage to the endothelium and is typically caused by high cholesterol levels, elevated blood pressure, diabetes, and smoking. Activated endothelial cells then express various adhesion molecules and cytokines, which promote the migration of circulating leukocytes, such as monocytes, T lymphocytes, and dendrites, into the subendothelial space of the artery. Once recruited, the monocytes develop into macrophages, which then ingest cholesterol and modified lipids, becoming foam cells on the basis of their appearance. This is the earliest detectable stage of atherosclerosis and is termed a fatty streak. Impaired emigration of the foam cells leads to their accumulation in the subendothelial space. Eventually, these cells die by necrosis, leaving a core of crystalline cholesterol and cell debris. Smooth muscle cells migrate into the area and deposit extracellular matrix proteins to form a protective cap over the lesion. Instability in the cap, arising from expression of matrix metalloproteinases, for example, may lead to rupture and subsequent thrombus formation[1].

Persistent and prolonged inflammatory mechanisms are believed to underpin atherosclerosis[1,2,3,4]. Examination of the developing lesion indicates up-regulation of such inflammatory proteins as interleukin-6 (IL-6), tumor necrosis factor (TNF)-α, and vascular cell adhesion molecule-1 (VCAM-1)[5,6]. Knockdown of proinflammatory proteins, such as intercellular adhesion molecule-1 (ICAM-1)[7], VCAM-1[8], TNF-α[9], and IL-18[10], in mouse models of atherosclerosis reduces the formation of atherosclerotic plaques and inhibits the progression of the disease.

Activation of the immune system is an important component of the chronic inflammatory state in atherosclerosis[1,11]. The immune system is comprised of two parts: the innate and adaptive systems. The innate immune system is the first line of defense against invading organisms. Toll-like receptors (TLRs) play a key role in the innate system by recognizing evolutionarily conserved structures in pathogens. TLRs can be divided into two groups based on their localization. TLR1, TLR2, TLR4, TLR5, TLR6, and TLR11 are found within cell walls and recognize components of microbial membranes. TLR3, TLR7, TLR8, and TLR9 are expressed internally in compartments, such as the endoplasmic reticulum, endosomes, and lysosomes, where they bind to nucleic acids of microbial or viral origin[12].

## TLRs AND ATHEROSCLEROSIS

Several lines of evidence suggest that TLRs are involved in atherosclerosis. TLR1, TLR2, and TLR4 are up-regulated in human atheroma with active NF-κB colocalizing with TLR2 and TLR4 in the plaque[13]. Excised and cultured human carotid plaques stimulated with CpG DNA, a TLR9 ligand, secreted interferon (IFN)-α and stimulation with lipopolysaccharide (LPS), a TLR4 ligand, induced secretion of TNF-α[14]. Various mouse models have also been used to investigate the possible role of TLRs in atherosclerosis. TLR4 is activated by Gram-negative bacterial LPS via the lipid-binding membrane protein CD14. Once LPS is bound to CD14/TLR4, the complex acquires various signaling adaptor molecules, including myeloid differentiation factor 88 (MyD88). Deletion of MyD88, but not that of CD14, reduced early lesion development in hyperlipidemic mice. This was due to a decrease in macrophage recruitment into the artery[15]. Apolipoprotein E (ApoE)–deficient mice lacking TLR4 displayed reduced aortic atherosclerosis, lower levels of circulatory proinflammatory cytokines, and decreased lipid content in the plaques[16]. Similarly, progression of atherosclerosis in LDLr-/- mice lacking TLR2 is reduced with concomitant decreases in macrophage recruitment and proinflammatory cytokine levels[17,18]. Interestingly transplantation of bone marrow cells without TLR2 had no effect on disease progression, suggesting that an endogenous ligand was influencing the disease by activating TLR2 in cells not of bone marrow origin[18].

The TLR4 polymorphism Asp299Gly is associated with a decreased response to inhaled LPS[19]. Several studies have suggested that this polymorphism in the human *TLR4* gene is associated with atherosclerosis. A reduced risk of carotid artery atherosclerosis development[20] and the appearance of acute coronary events[21,22] have all been observed in association with the Asp299Gly polymorphism. This polymorphism also appears to act synergistically with statin treatment; in a study of 655 men with angiographically documented coronary atherosclerosis, pravastatin reduced the risk of cardiovascular events by about 1.6-fold, whereas in the 299G carriers, the reduction of risk was more striking at about 15-fold[22]. Holloway et al. also found that compared with 299Asp homozygotes, 299Gly carriers had a significantly lower risk of myocardial infarction only when they were receiving statins[23]. Inhibition of the LPS/TLR4 proinflammatory signaling pathway by statins appears to involve either Rho[24] or reduced expression of the TLR4 protein[25].

## TLR SIGNALING

Ligand binding to TLRs stimulates expression of various inflammatory molecules, including IL-6, TNF-α, and IL-1β[12,26,27]. Once activated, the TLR recruits adaptor proteins, including MyD88, TRIF, TRAM, and TIRAP, via TIR-domain interactions. MyD88, with the exception of TLR3, is common to all TLR signaling pathways and is involved in MAPK (JNK, ERK, p38α) and proinflammatory transcription factor (NF-κB, AP1, Elk1) activation. Mice lacking MyD88 do not activate MAPK and proinflammatory transcription factors in response to TLR2-, TLR5-, TLR7-, TLR8-, and TLR9-specific ligands[28,29,30,31,32,33]. TLR3 and TLR4 signal through a MyD88-independent pathway. Cells deficient in MyD88 do not produce inflammatory cytokines in response to LPS; however, activation of the transcription factor IRF3 and increased expression of type-I IFN is unaffected[34]. Similarly, polydI.dC, the TLR3 ligand, stimulates IFN expression in the absence of MyD88[35,36]. TRIF was subsequently identified as the adaptor molecule that regulated this MyD88-independent pathway[37,38]. The adaptors TRAM and TIRAP confer specificity; TRAM participates in the TRIF pathway of TLR4, but not that of TLR3[26], and TIRAP is involved in MyD88-dependent signaling for some TLRs (TLR1, TLR2, TLR4, and TLR6), but not others (TLR5 and TLR9)[12,39,40].

## TLR EXPRESSION IN CELLS OF THE ATHEROSCLEROTIC LESION

The cells of the atherosclerotic lesion (macrophages, lymphocytes, dendritic cells, smooth muscle cells, and endothelial cells) all express TLRs[41]. Macrophages express a large number of TLRs, including TLR1, TLR2, TLR4, TLR5, TLR6, TLR8, and TLR9[42,43]. Increased expression of TLR2 and TLR4 has been observed in monocytes from human subjects and ApoE-/- mice with coronary artery disease[44,45,46]. Once recruited into the lesion, these macrophage-borne TLRs are exposed to a wide array of ligands; subsequent activation of these TLRs is important in converting the macrophages into foam cells[47,48,49]. Dendritic cells, both myeloid and plasmacytoid, accumulate in the atherosclerotic plaque and enhance T-cell stimulation[50,51]. Similar to macrophages, a large repertoire of TLRs are expressed in both dendritic cell subsets[52,53,54,55]. The differentiation of naïve T lymphocytes into $T_H1$ or $T_H2$ cells, which are considered pro- or antiatherogenic, respectively, is affected by TLRs. Knockout of MyD88 switches differentiation of T cells from $T_H1$ to $T_H2$[56,57]. Smooth muscle and endothelial cells respond to TLR ligands by producing proinflammatory cytokines[58,59,60,61]. Arterial endothelial and smooth muscle cells respond to a wider range of TLR ligands than the corresponding venous cell types[59]. In low-density lipoprotein receptor-deficient (LDLr(-/-)) mice, TLR2 expression is only found in aortic endothelial cells within regions of disturbed blood flow. These regions are particularly prone to the development of atherosclerotic lesions[62]. Expression of the receptor is important for leukocyte infiltration, foam cell formation, and endothelial cell injury within these disturbed flow areas[63].

## TLR LIGANDS AND ATHEROSCLEROSIS

The realization that TLRs are involved in atherosclerosis has prompted the search for the ligands involved in disease progression. Both exogenous and endogenous ligands have been shown to activate TLRs and many have been implicated in atherosclerosis (summarized in Table 1).

### Exogenous Ligands

Pathogen-borne TLR ligands have been shown to encourage the development of atherosclerosis. Administration of the TLR4 ligand LPS into ApoE-/- mice or hypercholesterolemic rabbits promotes atherosclerosis[64,65]. Intraperitoneal administration of a synthetic TLR2/TLR1 agonist, Pam3CSK4, increased atherosclerosis in LDLr-/- mice[18]. Murine macrophages treated with CpG-ODN, a TLR9 ligand, and oxidized LDL developed into foam cells in a NF-κB– and IRF7-dependent mechanism[66].

Acute infections are associated with a transiently increased risk of cardiac events[67] and various epidemiological studies have implied a causal link between infections, both bacterial and viral, and atherosclerosis. To date, these studies have associated atherosclerosis with *Chlamydia pneumoniae*, *Helicobacter pylori*, *Porphyromonas gingivalis*, *Cytomegalovirus*, Epstein-Barr virus, *Herpes simplex*, and HIV[68,69,70,71]. A potential mechanism whereby *Chlamydia pneumoniae* promotes atherosclerotic

**TABLE 1**
**TLR Ligands Implicated in Atherosclerosis**

| TLR | Ligand |
|---|---|
| TLR 2 | Peptidoglycan<br>Apo CIII<br>Oxidized LDL<br>HSP60<br>HMGB1<br>Hyaluronan |
| TLR3/9 | Viral dsDNA<br>CMV |
| TLR4 | LPS<br>Tenascin C<br>Fibrinogen<br>Fibronectin EDA<br>Heparin sulfate<br>HSP60<br>β-Defensin 2<br>Modified LDL<br>HMGB1<br>Hyaluronan |

lesion development has been elucidated using peritoneal macrophages from wild-type, TLR2, TLR3, TLR4, MyD88, TRIF, and IRF3 knockout mice. Live and UV-killed *C. pneumoniae* promoted foam cell formation in the presence of oxidized LDL in wild-type and TLR3 knockout macrophages. The genetic absence of TLR2, TLR4, MyD88, TRIF, and IRF3 all reduced foam cell formation[47]. If infection is a causal factor for atherosclerosis, the use of antibiotics would be expected to reduce the number of cardiac events. However, several studies investigating this hypothesis failed to show a positive effect deriving from antibiotic therapy[72,73,74,75]. Similarly, injection of pathogens into mice has had contrasting effects; acceleration of the progression of atherosclerosis was observed in some studies[76,77,78,79,80], whereas no effect was seen in others[81,82].

## Endogenous Ligands

A large number of structurally diverse endogenous molecules have been described as TLR ligands.

In the context of atherosclerosis, perhaps the most pertinent of the proposed endogenous TLR ligands are the modified LDLs. LDL oxidation and glycation are both important for atherogenesis[83,84,85]. Oxidized LDL promotes inflammatory signaling through a TLR4 and TLR6 heterodimer, the assembly of which is regulated by the scavenger receptor CD36[86]. This mechanism also mediates amyloid-β inflammatory signaling[86]. There is a positive correlation between the levels of the advanced glycation end product of LDL (AGE-LDL) and the risk of coronary heart disease[87]. In one study, AGE-LDL promoted inflammatory cytokine synthesis in human coronary endothelial cells and macrophages. Coimmunoprecipitation and antibody-blocking experiments indicated a role for TLR4, RAGE (receptor-for-advanced-glycation-end products), and CD36. The TLR4 signaling pathway induced by AGE-LDL included the MAPK (JNK, ERK1, and p38α) as well as the AP1, Elk1, and NF-κB transcription factors. Despite this signaling pathway being similar to that stimulated by LPS, significantly, the two polymorphisms in TLR4, Asp299Gly and Thr399Ile, which attenuate TLR4 responses to LPS, had no effect on AGE-LDL signaling[85]. Minimally oxidized LDL (mLDL), LDL modified by 12/15-lipooxygenase, has been heavily and well characterized as a TLR ligand. mLDL is believed to represent an early form of oxidized LDL and knockout studies have stressed the importance of 12/15-lipooxygenase in atherosclerosis[88,89]. The biologically active components of mLDL are cholesterol ester hydroperoxides[90]. Initially, mLDL was found to bind to CD14 and activate TLR4 in macrophages[91]. Subsequent studies highlighted that unlike LPS, the main effect of the binding of mLDL to the TLR4 complex was cytoskeletal changes and not proinflammatory gene expression[91,92,93]. Actin polymerization and membrane ruffling in response to mLDL required the recruitment of Syk to the TLR4 complex and the subsequent activation of various proteins, such as paxillin, Rac, Cdc42, and Rho, involved in cytoskeletal regulation. PI3K activation was also required, but this was independent of TLR4[94,95]. This membrane ruffling facilitated the uptake of LDL, modified or otherwise, into the macrophage by pinocytosis. In addition to cytoskeletal changes, mLDL has also been shown to stimulate the formation of reactive oxygen species (ROS) within macrophages in a TLR4-dependent and MyD88-independent manner. ROS generation resulted from NAPDH2 activation, a process that required Syk, phospholipase C-γ and PKC. In response to elevated ROS levels, proinflammatory proteins, such as IL-1β, IL-6, and RANTES, were expressed[92].

Certain oxidized phospholipids have been suggested as TLR4 ligands. Oxidized phospholipids can be generated during cell apoptosis and necrosis, as well as the oxidation of LDL. Accumulation of oxidized phospholipids within the plaque induces the expression of various proinflammatory proteins[96,97]. Oxidized 1-palmitoyl-2-arachidonoyl-sn-glycero-3-phosphorylcholine (ox-PAPC) stimulates IL-8 synthesis in aortic endothelial cells. Dominant-negative constructs and antisense oligonucleotides were used to demonstrate that TLR4 is the ox-PAPC receptor mediating IL-8 transcription. Phosphatidylinositol-specific phospholipase C pretreatment inhibited the effect of ox-PAPC, indicating that a glycosylphosphatidylinositol (GPI)-anchored protein was also necessary. This was not CD14, the GPI-anchored protein that is the TLR4 coreceptor for LPS; CD14-neutralizing antibodies had no effect on ox-PAPC–induced IL-8 transcription and there was no augmentation with the addition of soluble CD14[98]. Intriguingly, ox-PAPC also inhibits LPS activation of TLR4; in endothelial cells, LPS stimulation of MCP-1 and IL-8 secretion was inhibited by ox-PAPC[99]. Ox-PAPC interferes only with TLR4 and TLR2 signaling[99,100]. Two mechanisms have been proposed: prevention of TLR translocation to a lipid raft necessary for signaling or competition between ox-PAPC and TLR for binding to accessory proteins.

Apo-CIII directly activates monocytes, enhancing their adhesion to endothelial cells and stimulating VCAM-1 expression[101]. Apo-CIII binds with high affinity to TLR2. Deletion of TLR2 prevented Apo-CIII activation of monocytes[102].

Inflammation substantially increases the expression of serum amyloid A, and a positive correlation exists between serum amyloid A levels and cardiovascular risk[103]. Serum amyloid A can displace Apo AI from the surface of HDL particles. This reduces the antiatherogenic effects of HDL[104]. Additionally, solid-phase binding assays have indicated that serum amyloid A binds to TLR2 and, furthermore, in mouse macrophages lacking TLR2, changes in cytokine gene expression induced by serum amyloid A were reduced when compared to wild-type macrophages[105].

LPS is comprised of three parts: the O-antigen, the core oligosaccharide, and Lipid A. The toxicity of LPS is caused by Lipid A[106]. Due to the saturated fatty acid nature of the Lipid A moiety, it has been postulated that nonbacterial saturated fatty acids may themselves also be TLR4 agonists. Indeed, the saturated fatty acid lauric acid stimulated NF-κB and COX-2 expression in macrophages in a TLR4-dependent manner[107,108]. This signaling pathway is dependent on Akt, as expression of a dominant-negative Akt inhibited MyD88-induced NF-κB activation[109].

LPS-induced proinflammatory cytokine production in macrophages is suppressed by the consumption of n-3 polyunsaturated fatty acids[110]. Docosahexaenoic acid (DHA) and eicosapentaenoic acid (EPA), the major n-3 polyunsaturated fatty acids in fish oil, suppressed LPS activation of a COX-2 reporter stably transfected in the murine monocytic cell line RAW 264.7. DHA was found to inhibit a constitutively active TLR4, but not a constitutively active MyD88, indicating that inhibition was likely to be at the receptor level and not on downstream signaling proteins[111]. Similarly, LPS activation of dendritic cells is also inhibited by DHA[112]. The concept of dietary fatty acids modulating TLR activity is clearly an important issue in inflammatory diseases. Fatty acid–induced insulin resistance, which promotes atherosclerosis, has been linked to TLR4[113]. Free fatty acids, the levels of which increase in obesity, are believed to cause insulin resistance *in vivo* by activating proinflammatory signaling pathways in adipocytes[114,115,116,117]. TLRs activate NF-κB and JNK pathways, which are capable of inducing insulin resistance[115,116]. Shi et al. demonstrated that palmitate and oleic acid, two common free fatty acids, were capable of activating TLR4. These free fatty acids induced the release of IL-6 and TNF-α from isolated mouse adipocytes, an effect not observed in adipocytes from TLR4-/- mice or adipocytes treated with a TLR4 shRNA. Suppression of insulin-mediated glucose uptake by lipid infusion, which mimics insulin resistance, was reduced in TLR4-/- mice[113]. TLR activation by fatty acids may be sex specific; TLR4 knockout afforded some protection from a high-fat-diet–induced insulin resistance only in female mice[113,118].

The heat shock protein HSP60, a protein implicated in atherosclerosis[119], has been described as a TLR4 ligand. HSP60 elicited strong inflammatory TNF-α cytokine release from C57BL/6 and C3H/HeN macrophages. However, macrophages from C3H/HeJ mice, which possess a mutation rendering TLR4 inactive, did not release any TNF-α in response to HSP60 treatment[120].

The heat shock protein gp96 activates dendritic cells to produce proinflammatory proteins. This effect is reduced in dendritic cells derived from C3H/HeJ mice and inhibited still further in dendritic cells from C3H/HeJ/TLR2(-/-) animals[121]. Intriguingly, gp96 has been implicated in retention of TLR2 and TLR4 within the Golgi[122]. In human coronary artery endothelial cells, TLR4 functions intracellularly, with the LPS-binding protein aiding the internalization of LPS[123].

High-mobility group box protein 1 (HMGB1) was originally identified as a DNA-binding protein, but has subsequently been shown to act as a proinflammatory molecule[124,125]. HMGB1 is secreted from macrophages and smooth muscle cells within the atherosclerotic plaque[126,127]. Receptors identified for HMGB1 include RAGE[128] and both TLR2 and TLR4[129,130,131]. Significant cross-talk exists between the HMGB1-stimulated RAGE and TLR2/4 signaling pathways. RAGE activates Rac1, CDC42, Ras, and p38 MAPK. TLR2/4, which utilize MyD88 and IRAK, and also stimulate Rac1 and p38 MAPK. The common end point of both the RAGE and TLR2/4 pathways is the activation of NF-κB[132]. Inflammatory signaling by HMGB1 is self-sustaining by virtue of NF-κB–binding sites within the RAGE and TLR2 promoters[133,134]. This type of positive feedback mechanism may amplify inflammation in atherosclerosis.

Cellular fibronectins, which contain an alternatively spliced exon encoding a type-III repeat extra domain A (EDA), are produced during inflammation and may have tissue-remodeling properties[135]. Deletion of the EDA exon reduces atherosclerosis in ApoE-/- mice[136]. EDA-containing fibronectins have similar effects as LPS and indeed a recombinant EDA activated TLR4. EDA activity was heat dependent, unlike LPS, and the effect persisted in the presence of E5564, a LPS antagonist[137].

Fibrinogen is a plasma glycoprotein that is converted into fibrin during blood coagulation. Elevated fibrinogen levels are associated with an increased risk of cardiovascular disease[138]. Macrophages secrete chemokines in response to fibrinogen and several lines of evidence indicate that this effect is TLR4, and perhaps additionally TLR2, dependent. Smiley et al. found that the stimulation of macrophage chemokine secretion by fibrinogen was heat sensitive, unaffected by the LPS antagonist polymyxin B, and stimulation failed to occur in macrophages from C3H/HeJ mice[139]. Macrophage responses to fibrinogen are also blocked with an antibody to CD14, the TLR4 coreceptor[140]. Cultured podocytes also release chemokines such as MCP-1 in response to fibrinogen. Knockdown of either TLR2 or TLR4 siRNA has been observed to inhibit this effect[141,142]. Overexpression of a dominant-negative MyD88 in neonatal cardiomyocytes reduced fibrinogen-stimulated NF-κB activation[143]. In HEK293-CD14-MD2 cells expressing TLR4, fibrinogen induced the phosphorylation of the MAPK Erk1, p38α, and JNK, and activated the transcription factors AP1, NF-κB, and Elk-1[144]. Intriguingly, in this system, the TLR4 mutations that decrease responsiveness to LPS, Asp299Gly and Thr399Ile, increased the response to fibrinogen[144].

Glycosaminoglycans have been implicated in atherosclerosis[145,146,147,148] and, indeed, three members of the glycosaminoglycan family, hyaluronan, heparan sulfate, and versican, have been shown to be TLR ligands. Hyaluronan (HA), upon injury, is degraded into various low-molecular-weight fragments that activate inflammatory processes in a variety of cell types, including endothelial cells, dendritic cells, and macrophages[149,150,151,152,153,154]. Both TLR2 and TLR4 have been implicated as receptors for these low-molecular-weight HA fragments (low-HA). In the study of Scheibner et al., low-HA were found to stimulate inflammatory protein expression in a TLR2-dependent pathway that included MyD88, IRAK, TRAF6, PKCζ, and NF-κB[155]. TLR2 was identified as the receptor using stable transfected HEK293 cells[155]. Macrophages from C3H/HeJ mice responded to low-HA in the same fashion as C57BL/6 macrophages that have functional TLR4[155]. This is in contrast to the study of Termeer et al., where low-HA was unable to stimulate dendritic cells from C3H/HeJ and C57BL/10ScCr mice, which lack functional TLR4. Furthermore Termeer et al. reported that dendritic cells from TLR2-deficient mice had a normal response to low-HA[156]. A further study using macrophages found that although low-HA stimulation of cytokine synthesis was reduced in TLR4-/- or TLR2-/- cells, it required removal of both receptors to completely remove the effect[153]. Heparan sulfate is rapidly shed from basement membranes and cell surfaces in response to inflammation[157,158,159]. Systemic inflammatory response syndrome (SIRS) mainly occurs in response to trauma or surgery. The syndrome resembles sepsis, which is triggered by bacterial LPS acting on TLRs. SIRS can occur in the absence of infection, however, administration of soluble heparan sulfate into mice is sufficient to induce a SIRS-like response. This did not occur in mice lacking TLR4[160]. Dendrites mature in response to heparin sulfate, an effect that was abrogated when TLR4 was mutated or inhibited[161]. Stimulation with heparin sulfate also induced dendritic cells to secrete proinflammatory cytokines, such as IL-6 and TNF-α; an effect that was TLR4 dependent[162]. Versican has been implicated in lipid retention, inflammation, and thrombosis[163]. Conditioned media from Lewis lung carcinoma cells stimulates macrophages to produce IL-6 via TLR2 and the TLR2 coreceptor TLR6. Purification of the conditioned media identified versican as the ligand activating TLR2[164].

β-Defensins are a family of small mammalian antimicrobial peptides that prevent the colonization of epithelial surfaces by bacteria[165]. Murine β-defensin-2 has been implicated as a ligand for TLR4. Bone marrow–derived dendritic cells, when treated with recombinant β-defensin-2, displayed the maturation markers B7.2, CD40, and MHC class II. Pretreating the recombinant β-defensin-2 with proteinase K or by boiling prevented dendritic cell maturation; neither treatment had any effect on LPS-induced maturation. Additionally, immature dendritic cells from C3H/HeJ and C57BL/10ScNcr mice did not mature in response to β-defensin-2. Taken together, this evidence indicates that maturation of dendritic cells by β-defensin-2 is TLR4 dependent[166]. In addition to TLR4-dependent maturation, β-defensin-2 was found to promote an atypical form of cell death in dendritic cells that was caspase independent. This atypical cell death was dependent on up-regulation of plasma membrane–associated TNF-α and TNFR2. Increased TNF-α expression results from TLR4 activation. Such elimination of activated antigen presenting cells may act to limit inflammation[167].

TLR9 is activated by unmethylated CpG dinucleotides present in bacterial DNA[168] as well as chromatin-IgG complexes derived from the host organism[169,170]. CpG DNA activation of TLR9 has been shown to induce foam cell formation[171,172,173]. Currently, there are no studies indicating whether TLR9 activation by chromatin-IgG complexes would promote atherosclerosis. However, self-activation of a TLR implicated in atherosclerosis is a potentially interesting area of study.

## Cautionary Tales

Clearly, in studies investigating the potential of endogenous molecules as TLR activators, there is always the possibility of confounding results arising from contamination by microbial agents. Most of the atherogenic endogenous TLR ligands described above activate either TLR2 or TLR4. Considering that LPS will stimulate TLR4 at concentrations in the pg/mL range[174], contamination, however slight, is an important issue[175]. For example, the identification of C-reactive protein and HSP70 as TLR ligands has been described as artifacts arising from LPS contamination[176,177]. Various assays exist that are used by researchers to show that their preparations are not contaminated with LPS. The Limulus amebocyte lysate (LAL) assay is widely used to test for endotoxin or LPS contamination. However, LPS-binding protein and bactericidal/permeability–increasing protein inhibit LPS activity in the LAL assay[178]. This would suggest that any endogenous ligand that also binds to LPS would have a similar effect on the LAL assay[175]. Polymyxin-B is an antibiotic that is used to clear endotoxin contamination. The ability of polymyxin-B to inhibit the effects of LPS is dependent on the bacterial origin of the LPS. Whereas polymyxin-B will strongly inhibit LPS from *Escherichia coli* and *Acinetobacter calcoaceticus*, it had little effect on LPS derived from *Neisseria meningitidis*[179]. In a laboratory setting, *E. coli* would perhaps be the most likely microbial contaminant of an endogenous ligand preparation because of their widespread use in molecular biology. The other most widely used assay is heat treatment, as this is believed to have no effect on LPS activity. However, this is dependent on the LPS concentration. Tsan and Gao identified that LPS heat inactivation can be readily observed at LPS concentrations of 1 ng/mL. Considering the potency of LPS to stimulate cytokine synthesis and the likelihood of LPS contamination to be below 1 ng/mL, the authors noted the necessity of comparing the heat sensitivity of LPS at the same concentration as that present in the endogenous ligand preparation[180].

Despite the necessary caution, there are grounds for optimism regarding endogenous TLR ligands. Truly germ-free mice, devoid of any bacterial, viral, or fungal agents, develop atherosclerosis at the same rate as animals exposed to normal levels of microbial organisms[181]. Considering the importance of TLRs in the development of atherosclerosis, this would suggest that endogenous TLR ligands exist. Furthermore, bone marrow transplantation experiments indicate that bone marrow–derived cells are not involved in the atheroprotective effects of a TLR2 knockout in mice fed a high-fat diet, but were necessary for the atherogenic response to the exogenous TLR2 agonist Pam3[18]. Additionally, several of these endogenous ligands activate TLR signaling pathways in a fashion that markedly differs from LPS. Activation of TLR4 by mLDL predominantly affects the cell cytoskeleton and not the expression of inflammatory proteins like LPS[91,92,93]. Indeed, even in signaling molecules activated by both mLDL and LPS, there are clear differences. ERK phosphorylation was rapid with mLDL stimulation, but significantly slower with LPS. Phosphorylation of c-Jun in response to LPS is IKKε dependent and JNK independent; however, mLDL-induced c-Jun phosphorylation is dependent on JNK[182]. Similarly, the TLR4 D299G and T399I substitutions, which significantly reduce the ability of LPS to activate the receptor and so produce cytokines, had no effect on AGE-LDL stimulation of cytokine production through TLR4[85], and increased the ability of fibrinogen to stimulate TLR4[144].

## CROSS-TALK BETWEEN TLRS AND OTHER RECEPTORS

HMGB1, as discussed above, promotes inflammation via significant cross-talk between TLR2/4 and RAGE. Other examples exist whereby TLR signaling is modified by other receptors. The atheroprotective effect of sphingosine 1-phosphate occurs via attenuation of TLR2 signaling by the sphingosine 1-phosphate receptors class 1 and 2[183]. Liver X receptors (LXRs) negatively modulate TLR signaling pathways[47,66,184]; foam cell formation in response to either the TLR9 ligand CpG ODN or *Chlamydia pneumoniae* was inhibited by LXR agonists[47,66]. However, long-term treatment of macrophages with LXR agonists increased the response of TLR4 to LPS[185]. Peritoneal macrophages deficient in either ABCA1 or ABCG1, important cholesterol transporters in macrophages, have enhanced expression of proinflammatory gene expression. This increased inflammatory gene expression was abolished in macrophages lacking TLR4 or MyD88/TRIF. ABCA1 and ABCG1 deletion enhanced the response of macrophages to TLR2, TLR3, and TLR4 ligands; in the case of TLR4, this is presumably due to the observed increase in cell surface concentration of the receptor. These findings indicate that promotion of cholesterol efflux by HDL and Apo AI will also attenuate TLR signaling[186].

## CONCLUSION

TLRs, key regulators of the innate immune response, produce inflammatory proteins that can promote atherosclerosis. Pathogenic organisms have been implicated in atherosclerosis and may activate TLRs residing on cells within the atherosclerotic lesion. Currently, due to the lack of a positive effect from antibiotic therapy, the question of what role microbial agents play in atherosclerosis remains open. Various endogenous proteins, which promote or sustain progression of the disease, have also been implicated as TLR ligands. The discovery of these endogenous TLR ligands provides novel insights into the pathogenesis of coronary artery disease. However, much remains to be understood. The exact nature of these endogenous proteins as TLR ligands and how they interact during the development of atherosclerosis need to be addressed. However, once these issues are clarified, modulating the effects of endogenous ligands on TLRs has a clear therapeutic benefit in the treatment of atherosclerosis.

## ACKNOWLEDGMENTS

This work was supported by British Heart Foundation. We would like to thank Jose A. Gomeź for critical reading of the manuscript.
